# Machine Learning for Predicting Dental Caries Presence and Severity: An Age-Cohort Perspective from a Nationally Representative Survey in Brazil

**DOI:** 10.1159/cre/abuag004

**Published:** 2026-10-10

**Authors:** Jessye Melgarejo do Amaral Giordani, Orlando Luiz do Amaral Junior, Maria Laura Braccini Fagundes, Juliana Balbinot Hilgert, Fernando Neves Hugo

**Affiliations:** a Federal University of Santa Maria, Santa Maria, Brazil; b Federal University of Rio Grande Do Sul, Porto Alegre, Brazil; c Department of Epidemiology and Health Promotion, New York University College of Dentistry, New York, NY, USA

**Keywords:** Artificial intelligence, Cariology, Machine learning, Epidemiology, Public health planning, Risk stratification

## Abstract

**Introduction:**

Although preventable, dental caries remains a global public health challenge due to its multifactorial nature and the deep influence of socio-structural determinants. This study evaluates the performance of artificial intelligence (AI) models as decision-support frameworks for population-level risk stratification, screening, and public health planning.

**Methods:**

We analyzed a representative sample (*N* = 23,726) from the SB Brasil 2023 national survey, stratified into three age cohorts: children (12 years), adolescents (15–19 years), and adults (35–44 years). Eleven predictors, encompassing socioeconomic (wealth score via PCA, household density, race, water access), psychosocial (self-rated oral health status), behavioral, and clinical domains, were used to predict caries presence (≥1 untreated decayed tooth) and severity (DMFT index regression). To account for overdispersion and the high frequency of zero counts, we utilized advanced count-based regressions, including Zero-Inflated Negative Binomial (ZINB) and Negative Binomial models. Performance was assessed using AUC, mean absolute error (MAE), and decision curve analysis (DCA) via 5-fold cross-validation.

**Results:**

Caries prevalence increased progressively with age (36.8–52.7%). The non-clinical triage models demonstrated significant net benefit in DCA across all groups, effectively identifying high-risk individuals for prioritization without requiring clinical examination. For severity prediction, the ZINB model identified structural signals of inequity, particularly in late adolescence (15–19 years), where socio-structural determinants explained a meaningful proportion of clinical variance (*R*2 = 0.1010; MAE = 2.79). Across all stages, SHAP analysis reinforced that wealth score and household density are the primary structural predictors of caries risk.

**Conclusion:**

AI provides a feasible framework for population-level risk stratification and public health planning. The findings suggest that AI-driven frameworks should be utilized for managerial triage and resource allocation, enabling proportional universalism by identifying high-risk clusters through structural determinants rather than individual-level clinical diagnostics.

## Plain Language Summary

Tooth decay remains a major health problem worldwide, deeply influenced by social factors like wealth, race, and living conditions. This study used artificial intelligence (AI) to evaluate how these computer programs can help health managers identify at-risk populations and plan dental services more effectively. We analyzed data from over 23,000 people in Brazil, focusing on 3 groups: 12-year-olds, teenagers (15–19 years), and adults (35–44 years). A key feature of this new analysis was the development of a “Non-Clinical Triage” model. This model uses only social and economic information – such as family wealth and housing conditions – to predict risk without requiring a physical dental examination first. Our results confirmed that tooth decay increases significantly with age. We found that AI models are highly reliable at spotting those with the highest disease burden, especially among adults. By using advanced mathematical models that account for the specific patterns of dental health data, we showed that socio-structural factors like family wealth and household density are the most powerful predictors of tooth decay throughout a person’s life. In conclusion, AI provides a valuable tool for public health systems to prioritize care and allocate resources more fairly. Rather than just making individual diagnoses, these frameworks allow health managers to identify “high-risk clusters” based on the social conditions in which people live. This approach supports a more efficient dental health system that addresses the root causes of oral health inequities.

## Introduction

Dental caries remains a significant global health paradox. Although it is a preventable disease with well-known etiologic factors and established prevention methods, it continues to be the most prevalent disease globally, affecting billions of individuals across all age-groups [1]. This persistence is driven by a complex interplay of high exposure to fermentable carbohydrates, a well-adapted oral microbiome, and social normalization of tooth decay as an unavoidable part of the lifespan [2].

In recent decades, the scientific focus in cariology has shifted from a purely physico-chemical and microbiological perspective toward a more comprehensive understanding of behavioral, social, and economic determinants [3, 4]. This transition underscores the complex nature of dental caries, where structural inequities, such as race, wealth, and access to basic infrastructure, act as fundamental drivers of oral health outcomes [5, 6]. Consequently, effective caries management requires innovative and creative approaches to move beyond traditional diagnostic pathways and translate research findings into viable, effective population-level public health solutions [7].

The emergence of artificial intelligence (AI) and machine learning offers a transformative opportunity for public health surveillance and risk stratification [8]. By processing high-dimensional epidemiological data, such as the nationally representative SB Brasil 2023 survey, machine learning models can function as decision support frameworks for public health, providing population-level risk assessments and improving screening accuracy at a managerial scale [9–11]. In nations like Brazil, where oral health inequities are deeply rooted in socio-structural factors, the use of machine learning to drive managerial decisions, such as prioritizing care for vulnerable populations or optimizing resource allocation, is essential [12–14]. However, the successful integration of AI into dentistry depends on the transparency and interpretability of these models, ensuring that technology signals structural inequities rather than perpetuating existing biases [15, 16].

To our knowledge, no study has evaluated the performance of different machine learning algorithms in predicting dental caries presence and severity from an age-cohort perspective, while simultaneously evaluating the relative contribution of socioeconomic, demographic, psychosocial, behavioral, and clinical determinants. Existing research has predominantly focused on specific subsets of this scope, often limited to single age-groups or specific caries types. For instance, studies have utilized machine learning to classify root caries in adults [14] or to analyze risk factors in non-pediatric populations [17], yet these often exclude severity metrics and comprehensive age-cohort breadth. Similarly, pediatric-focused works have successfully incorporated early-life predictors [18, 19], but remain restricted to childhood, leaving a significant gap in the integration of lifespan epidemiology with dual-outcome modeling of presence and severity [20]. Therefore, this study aims to evaluate the performance of different machine learning algorithms in predicting dental caries presence and severity within an age-cohort framework, to identify critical determinants and provide a decision support framework for AI-driven population-level screening and public health planning.

## Methods

### Study Design and Data Source

This study utilized data from the SB Brasil 2023 (Brazilian Oral Health Survey), a national cross-sectional epidemiological survey coordinated by the Brazilian Ministry of Health [21]. The survey targeted the urban population residing in permanent private households across the entire national territory, providing a representative sample of the Brazilian population [21]. For this analysis, three age cohorts were selected, focusing on specific index ages and age- groups recommended by the World Health Organization (WHO), to investigate the caries burden across the lifespan: children (12 years old), adolescents (15–19 years old), and adults (35–44 years old), reflecting the multifactorial nature of the disease and its impact at different developmental stages [22]. The study was conducted in accordance with the Transparent Reporting of a multivariable prediction model for Individual Prognosis Or Diagnosis-Artificial Intelligence (TRIPOD + AI) statement [20], and the risk of bias was evaluated based on the PROBAST + AI tool [23]. The survey was approved by the National Research Ethics Committee (CONEP), and all participants or their legal guardians provided written informed consent.

### Sampling Strategy and Sample Size

The SB Brasil 2023 employed a complex, three-stage cluster sampling design across 55 strata [21]. The first stage involved the selection of primary sampling units (municipalities) stratified by geographic region and population size. The second stage consisted of the selection of census sectors, and the third stage involved the systematic selection of households [21]. For children (12 years), a single-stage cluster sampling was used (census sectors), while for adolescents (15–19 years) and adults (35–44 years), a two-stage process involved the selection of census sectors followed by household selection. The total planned sample size reached 50,800 interviews. In state capitals, the sample was fixed at 300 individuals for the 15–19-year and 35–44-year groups, and 250 for the 12-year group, while the interior strata included an additional 100 individuals per age-group. All analyses were performed using inverse probability weighting to account for unequal selection probabilities [21].

### Data Collection and Standardization

Data collection was conducted by trained oral health teams from the Brazilian National Health System (SUS) using tablets. Examiners underwent a rigorous standardization process, with calibration performed using the In-Lux method [24]. Examiners were required to achieve a Kappa coefficient >0.60 (indicating substantial agreement) relative to the consensus before certification. Clinical examinations were performed under natural light using a CPI probe and a plane dental mirror following WHO criteria [22].

### Outcomes (Caries Metrics)

Two distinct outcomes were generated from the original clinical data to support population-level decision support frameworks:

a) Caries presence (classification): derived from the “decayed” component of the DMFT index, this was transformed into a binary variable (1 for individuals with ≥1 untreated decayed tooth; 0 otherwise) to support population-level risk stratification.
b) Caries severity (regression): calculated as the DMFT index, representing the sum of decayed (D), missing due to caries (M), and filled (F) permanent teeth; this outcome was utilized to support managerial and public health planning needs.

### Predictive Scenarios and Feature Engineering

To evaluate the practical utility of machine learning in public health, we developed two modeling scenarios: (1) the full clinical model, incorporating all clinical and socio-behavioral predictors, and (2) the non-clinical triage model, which excludes variables requiring clinical examination (gingival bleeding and calculus), relying exclusively on sociodemographic, economic, and self-reported data to evaluate screening efficiency at the population level. Our predictor selection was adapted from the conceptual model of oral health determinants proposed by Peres et al. [1]. Predictors were grouped into the following domains:

(a) Socioeconomic (and demographic): biologic sex, self-reported race/color, access to piped water, household density, and wealth score (generated using principal component analysis of household assets) [25, 26]
(b) Psychosocial: self-rated oral health status (5-point Likert scale) [27]
(c) Access and behavioral: time since the last dental visit and private dental insurance status
(d) Clinical: orthodontic appliance use, gingival bleeding, and dental calculus

To maintain consistency across age-cohort comparisons, the same set of 11 predictors was applied to all groups.

### Statistical Analysis and Machine Learning Pipeline

The computational analysis was performed using Python (v3.12.x) within the Google Colab Pro environment, utilizing NVIDIA L4 GPU acceleration. The machine learning pipeline was implemented using pandas and scikit-learn [28]. Gradient boosting models were executed via the XGBoost library [29].

Considering that the DMFT index is an asymmetric, count-based outcome, we performed overdispersion diagnostics using the Cameron and Trivedi test. Upon confirming significant overdispersion (*p* < 0.001), we replaced traditional linear models with advanced count-based frameworks: Negative Binomial and Zero-Inflated Negative Binomial regressions. These were compared against nonparametric ensemble methods, including random forest [30] and XGBoost [29], to capture high-dimensional nonlinear interactions between socio-structural determinants.

To ensure generalizability and prevent overfitting, 5-fold cross-validation was implemented during the training phase. Final classification performance was assessed via area under the receiver operating characteristic curve and calibration curves [31]. The practical utility was evaluated via decision curve analysis (DCA), where the net benefit was calculated across a range of threshold probabilities [32]. For severity prediction, model performance was measured using the mean absolute error (MAE), which expresses, in DMFT units, the average magnitude of the difference between predicted and observed scores, with lower values indicating higher accuracy. Finally, predictor contributions were quantified using SHapley Additive exPlanations (SHAP) values [33], a game-theoretic approach that assigns each predictor a numerical contribution to a given prediction, allowing the relative importance and direction of effect of individual variables to be ranked and visualized.

## Results

### Sample Characteristics and Epidemiological Baseline

The final analytical sample comprised 23,726 individuals: 6,698 children (12 years), 8,041 adolescents (15–19 years), and 8,987 adults (35–44 years). Data cleaning procedures resulted in minimal exclusions (*n* = 51 total), ensuring high representativeness. Regarding the sample distribution (shown in Table 1), most participants across all age cohorts identified as non-white (black/brown/other), representing 60.5% of 12-year-olds, 62.3% of adolescents, and 58.3% of adults. Structural and economic factors were consistent across groups, with a vast majority having access to piped water (ranging from 81.3% to 83.6%) and over 86% lacking private dental insurance. In terms of psychosocial and behavioral factors, while most children (57.7%) and adolescents (52.7%) perceived their oral health as “good”, this was less frequent among adults (40.9%). Time since the last dental visit was balanced across cohorts, with approximately half of the participants reporting a visit within the last year. Notably, the adult cohort (35–44 years) was predominantly female (66.3%), and the use of orthodontic appliances was concentrated among adolescents (16.3%). Clinical proxies revealed that adults presented the highest mean values for both gingival bleeding (1.22 ± 1.86) and dental calculus (1.40 ± 1.82).

**Table 1.** Weighted descriptive characteristics of the study population across age cohorts based on demographic, socioeconomic, psychosocial, behavioral, and clinical predictors from the Brazilian National Oral Health Survey, 2023 (*N* = 23,726)

Variables	12-year-old (*n* = 6,698)	15–19-year-old (*n* = 8,041)	35–44-year-old (*n* = 8,987)
Categorical predictors (%)			
Biological sex (female)	49.0	49.8	66.3
Race/color (non-white)	60.5	62.3	58.3
Piped water (at least one room)	82.4	81.3	83.6
Self-rated oral health status (good)	57.7	52.7	40.9
Time since last visit (≤1 year)	49.4	52.0	49.6
Private dental insurance (no)	90.0	88.0	86.5
Orthodontic appliance (yes)	9.3	16.3	0.0
Quantitative predictors (mean±SD)			
Wealth score	0.08±1.96	0.08±1.90	0.26±1.81
Household density	1.79±0.87	1.73±0.89	1.56±0.73
Gingival bleeding	0.80±1.58	1.04±1.79	1.22±1.86
Dental calculus	0.53±1.23	0.88±1.58	1.40±1.82

All percentages and mean ± SD values are weighted estimates. Wealth score was derived via PCA of 11 household assets to ensure a stable socioeconomic gradient. Household density was calculated as the ratio of residents per bedroom. Gingival bleeding and dental calculus represent the mean number of positive sextants per individual.

The caries burden exhibited a progressive and significant increase across the three age cohorts studied. The weighted mean DMFT index rose from 1.67 (SD 2.54) in children aged 12 to 3.41 (SD 4.41) in adolescents (15–19 years), reaching a peak of 10.70 (SD 6.84) in the 35–44-year adult cohort. Correspondingly, the weighted prevalence of untreated caries was 36.85% (95% CI: 33.81–39.89) in the 12-year cohort, increased to 43.73% (95% CI: 40.98–46.48) in the 15–19-year group, and reached its highest level at 52.74% (95% CI: 49.82–55.66) among adults in the 35–44-year group. These findings underscore the cumulative nature of the disease and provide the epidemiological baseline for the predictive performance of the machine learning models.

### Disparities across Predictive Domains

The distribution of dental caries exhibited marked disparities across all predictive domains, reflecting a complex interplay of socioeconomic, structural, and behavioral factors. Regarding race and color inequity, individuals identifying as non-white experienced a higher disease burden across all age-groups; specifically, in the 15–19 y cohort, the prevalence of untreated caries was significantly higher among black adolescents (54.2%) compared to their white counterparts (33.5%). Structural determinants such as water sanitation also played a critical role as 12-year-olds without access to piped water presented a caries prevalence of 62.5%, nearly double the 35.5% observed in those with access, while higher household density was positively correlated with both DMFT scores and caries prevalence across all cohorts. Furthermore, access and behavioral factors significantly influenced outcomes; adolescents (15–19 years) who had not visited a dentist in the last year showed a significantly higher prevalence of untreated caries (58.3%) compared to those with a recent visit (36.6%), and the lack of private dental insurance was associated with increased prevalence (51.4% in the uninsured vs. 27.8% among the insured). Finally, subjective perception and clinical proxies served as strong biological sensors; for instance, adults (35–44 years) rating their oral health as “very bad” presented a mean DMFT of 14.5 (SD 6.84) compared to 7.8 in the “very good” group.

### Machine Learning Performance: Severity and Presence

Machine learning performance for identifying caries presence and severity showed a progressive trend across the lifespan. Regression analysis (shown in Table 2) utilizing advanced count models, specifically Zero-Inflated Negative Binomial and Negative Binomial regressions, properly captured the structural signal of caries severity. A predictive shift in model performance was identified during late adolescence (15–19 years), where socio-structural determinants explained 10.06% of the variance (*R*2 = 0.1006) with a MAE of 2.80. Explanatory power was lower in the other age-groups, with R2 values of 0.0291 at 12 years and 0.0186 for the 35–44 group. Across all age cohorts, these probabilistic models proved to be the most stable, consistently outperforming high-complexity ensemble algorithms such as XGBoost, which exhibited negative R2 values, suggesting a lack of generalizability for this specific count-based epidemiological outcome.

**Table 2.** Predictive performance metrics of seven machine learning regression algorithms for the assessment of dental caries severity (DMFT index) among children, adolescents, and adults, from the Brazilian National Oral Health Survey, 2023 (*N* = 23,726)

Age-group	Model	R2^a^	MAE^a^	R2^b^	MAE^b^
12 years	Linear regression	0.0291	1.7594	−0.0004	1.7861
	Ridge regression	0.0291	1.7594	−0.0004	1.7861
	ZINB	0.0048	1.7813	0.0127	1.7866
	NB	−0.0334	1.8013	−0.0052	1.8018
	Gradient boost	0.0081	1.7990	−0.0145	1.8194
	Random forest	−0.1046	1.8659	−0.1965	1.8936
	XGBoost (GPU)	−0.3156	1.9987	−0.3252	1.9878
15–19 years	Linear regression	0.1006	2.8005	0.0817	2.8302
	Ridge regression	0.1006	2.8005	0.0817	2.8302
	ZINB	0.1010	2.7951	0.0927	2.8162
	NB	0.1002	2.8036	0.0898	2.8147
	Gradient boost	0.0912	2.8056	0.0701	2.8200
	Random forest	0.0401	2.9674	−0.0765	3.1483
	XGBoost (GPU)	−0.1245	3.0685	−0.2526	3.2062
35–44 years	Linear regression	0.0186	5.6085	0.0115	5.5873
	Ridge regression	0.0186	5.6085	0.0115	5.5873
	ZINB	0.0064	5.6350	0.0014	5.6181
	NB	0.0090	5.6154	0.0032	5.6075
	Gradient boost	−0.0013	5.6861	−0.0284	5.6688
	Random forest	−0.0230	5.7330	−0.1258	5.8903
	XGBoost (GPU)	−0.2183	6.1607	−0.1694	5.8408

Negative R2 values indicate that the model performed worse than a horizontal line representing the mean.

*R*2, coefficient of determination; MAE, mean absolute error; DMFT, decayed, missing, and filled teeth index.

^a^ Clinical full model.

^b^ non-clinical triage model.

The clinical utility of the regression models was confirmed via DCA, where the AI frameworks provided a superior net benefit compared to default strategies (Fig. 1a). Explainability analysis through SHAP values (Fig. 1b) revealed that structural vulnerabilities, represented by the wealth score and household density, were the most influential factors in predicting higher DMFT counts, followed by self-rated health and clinical indicators.

**Fig. 1.**
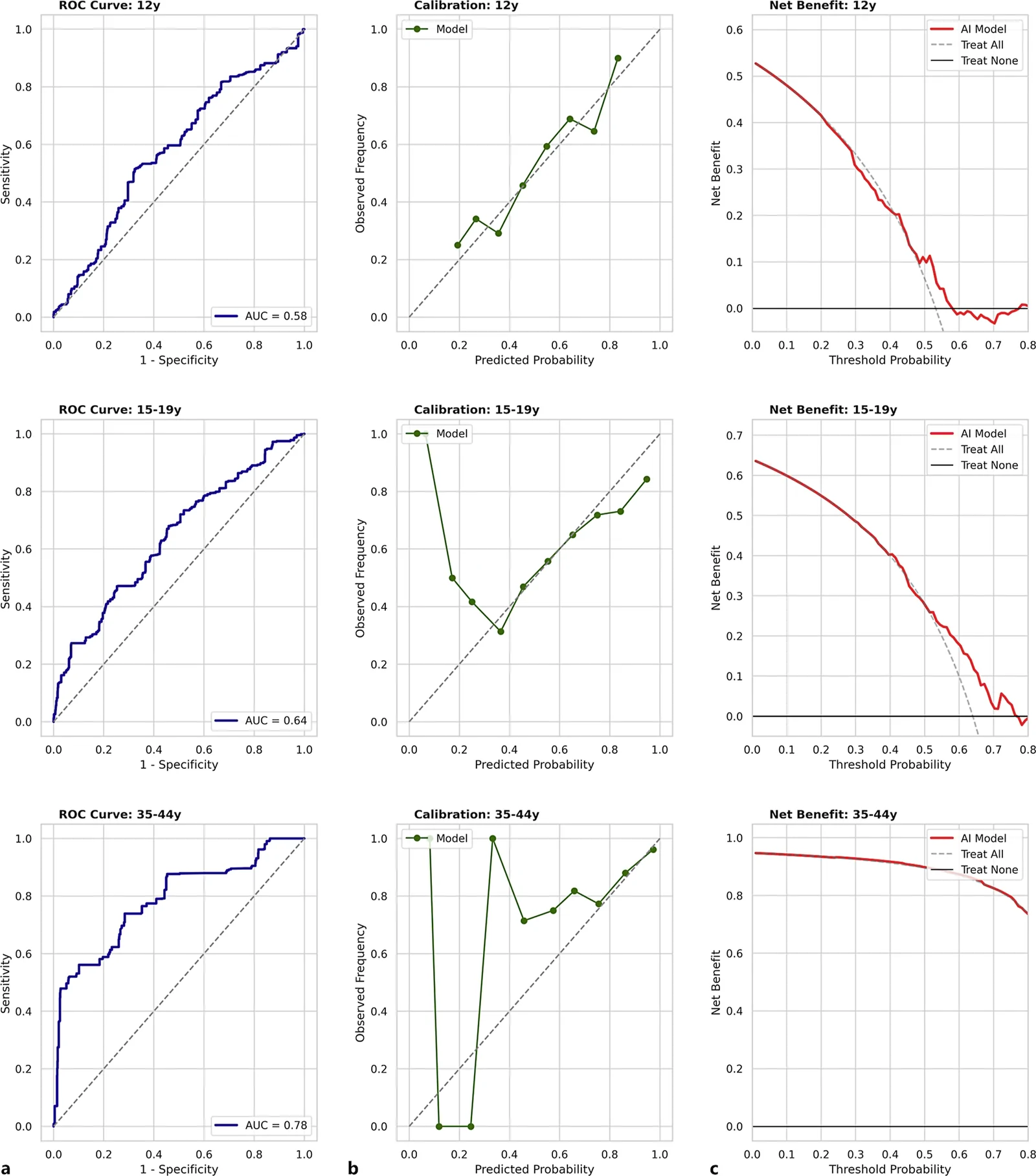
Clinical utility and determinant hierarchy for predicting caries severity (DMFT). **a** Decision curve analysis (DCA) demonstrating the positive net benefit of the AI severity model compared to default strategies. **b** SHAP summary plots ranking features by global importance, showing wealth score and household density as the primary structural drivers of severity across the lifespan.

The predictive performance of the classification models for dental caries presence demonstrated a clear progressive improvement as the age of the cohorts increased (shown in Table 3). In the 12-year-old cohort, discrimination power was relatively modest; random forest achieved the highest AUC (0.6139). For the 15–19 y group, predictive performance increased, with logistic regression emerging as a top performer in discrimination (AUC 0.6966). The highest predictive accuracy and discrimination were achieved in the 35–44-year adult cohort, where AUC reached 0.7418. Crucially, the non-clinical triage model maintained competitive performance across all cohorts, demonstrating that socio-behavioral and demographic data alone can effectively prioritize high-risk individuals for clinical examination. Across all cohorts, models generally exhibited higher specificity than sensitivity in younger ages, whereas the adult models showed a more balanced performance profile, reflecting the models’ increased capacity to identify true cases as the disease burden becomes more prevalent.

**Table 3.** Evaluation of five machine learning classification models for the predictive screening of dental caries presence (≥1 untreated decayed tooth) among children, adolescents, and adults from the Brazilian National Oral Health Survey, 2023 (*N* = 23,726)

Age-group	Scenario	Model	AUC	Acc	Sens	Spec	F1	PPV	NPV	Harm
12 years	Full clinical	Gradient boosting	0.5629	0.5585	0.5709	0.5444	0.5791	0.5876	0.5273	0.5573
		Logistic regression	0.5770	0.5649	0.5456	0.5868	0.5716	0.6003	0.5317	0.5654
		Random forest	0.5528	0.5250	0.4834	0.5723	0.5199	0.5624	0.4934	0.5241
		SVM (turbo)	0.5806	0.5681	0.5491	0.5897	0.5750	0.6034	0.5348	0.5686
		XGBoost (GPU)	0.5449	0.5340	0.5538	0.5115	0.5584	0.5632	0.5020	0.5318
	Non-clinical	Gradient boosting	0.5557	0.5546	0.5629	0.5451	0.5736	0.5846	0.5230	0.5539
		Logistic regression	0.5519	0.5424	0.5605	0.5218	0.5659	0.5714	0.5108	0.5405
		Random forest	0.5514	0.5176	0.4550	0.5888	0.5009	0.5572	0.4872	0.5133
		SVM (turbo)	0.5574	0.5546	0.5287	0.5842	0.5582	0.5912	0.5215	0.5550
		XGBoost (GPU)	0.5554	0.5630	0.5993	0.5217	0.5934	0.5876	0.5338	0.5578
15–19 years	Full clinical	Gradient boosting	0.6105	0.6506	0.8932	0.2200	0.7658	0.6702	0.5372	0.3531
		Logistic regression	0.6413	0.6445	0.8660	0.2512	0.7570	0.6724	0.5138	0.3895
		Random forest	0.6349	0.6529	0.8423	0.3167	0.7563	0.6863	0.5309	0.4603
		SVM (turbo)	0.6470	0.6368	0.9027	0.1649	0.7607	0.6573	0.4884	0.2788
		XGBoost (GPU)	0.6279	0.6436	0.7849	0.3929	0.7380	0.6965	0.5072	0.5237
	Non-clinical	Gradient boosting	0.5904	0.6429	0.8812	0.2201	0.7594	0.6672	0.5107	0.3522
		Logistic regression	0.6360	0.6433	0.8690	0.2428	0.7571	0.6707	0.5109	0.3795
		Random forest	0.6316	0.6381	0.7701	0.4037	0.7313	0.6962	0.4973	0.5300
		SVM (turbo)	0.6393	0.6431	0.9560	0.0878	0.7741	0.6503	0.5292	0.1608
		XGBoost (GPU)	0.6065	0.6291	0.7570	0.4021	0.7231	0.6920	0.4825	0.5252
35–44 years	Full clinical	Gradient boosting	0.7943	0.9505	0.9956	0.1375	0.9744	0.9541	0.6351	0.2417
		Logistic regression	0.7000	0.9433	0.9956	0.0011	0.9708	0.9473	0.0133	0.0021
		Random forest	0.6399	0.9242	0.9753	0.0045	0.9606	0.9464	0.0099	0.0089
		SVM (turbo)	0.7126	0.9474	1.0000	0.0000	0.9730	0.9474	0.0000	0.0000
		XGBoost (GPU)	0.6715	0.9335	0.9826	0.0476	0.9655	0.9490	0.1320	0.0908
	Non-clinical	Gradient boosting	0.7202	0.9382	0.9827	0.1369	0.9679	0.9535	0.3044	0.2402
		Logistic regression	0.7197	0.9433	0.9956	0.0011	0.9708	0.9473	0.0134	0.0021
		Random forest	0.6078	0.9136	0.9642	0.0013	0.9548	0.9457	0.0021	0.0027
		SVM (turbo)	0.7201	0.9474	1.0000	0.0000	0.9730	0.9474	0.0000	0.0000
		XGBoost (GPU)	0.5607	0.9150	0.9650	0.0134	0.9556	0.9463	0.0207	0.0264

SVM (Turbo) refers to the calibrated linear SVM model.

AUC, area under the ROC curve; Acc, accuracy; Sens, sensitivity; Spec, specificity; F1, F1 score; PPV, positive predictive value; NPV, negative predictive value; Harm, harmonic mean of sensitivity and specificity.

Model performance for caries screening improved with age, reaching an AUC of 0.78 in the adult cohort (Fig. 2a). Calibration plots indicated that the models were well-calibrated for low-to-moderate risk thresholds (Fig. 2b). Furthermore, DCA curves (Fig. 2c) demonstrate that utilizing the AI models for screening yields a higher net benefit, particularly for identifying high-risk individuals in the 35–44-year age-group.

**Fig. 2.**
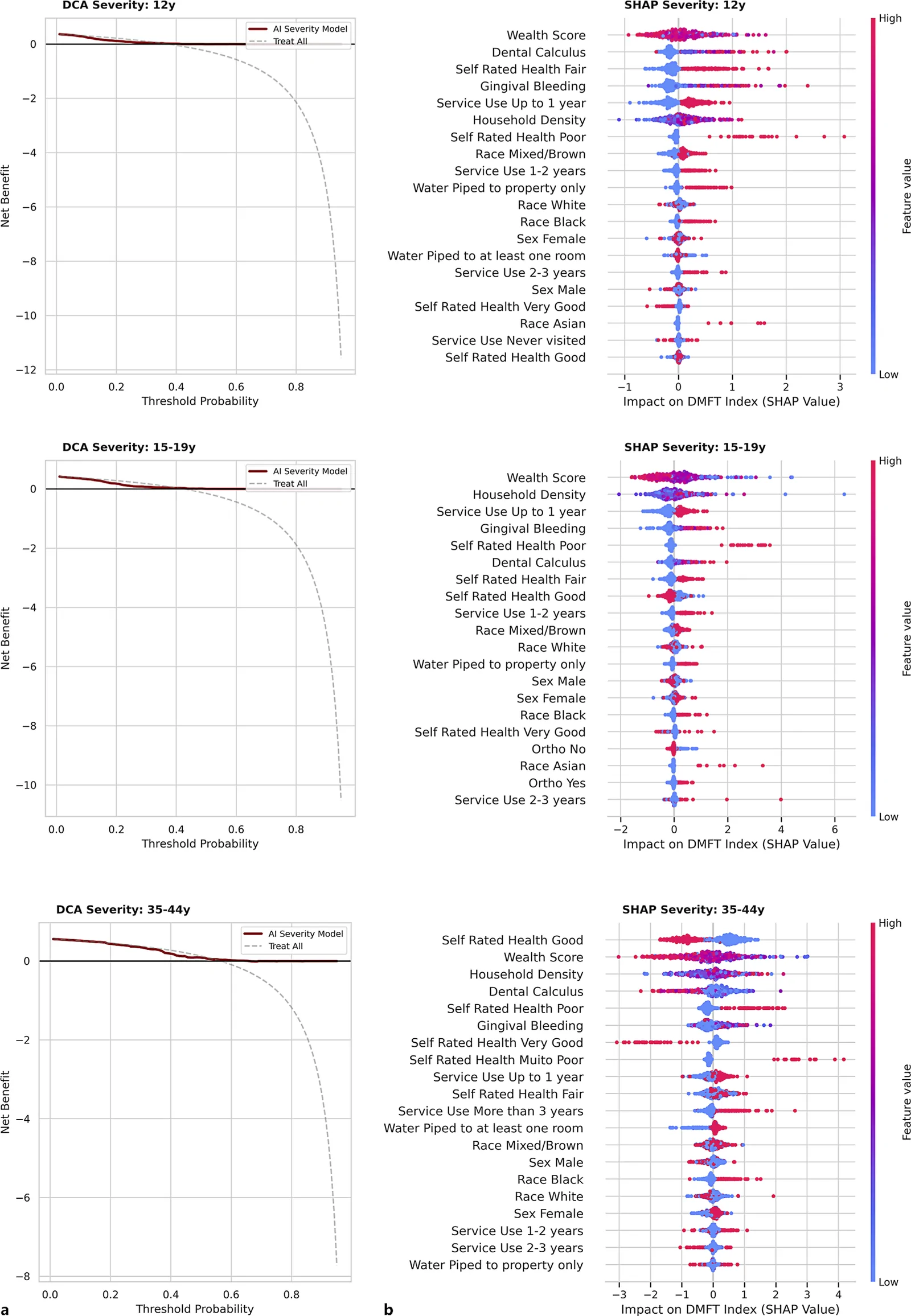
Performance, calibration, and utility metrics for caries presence classification (≥1 untreated decayed tooth). **a** Receiver operating characteristic (ROC) curves showing diagnostic performance across age cohorts. **b** Calibration plots comparing predicted probabilities against observed frequencies. **c** Net benefit curves illustrating the clinical utility of classification models for population-level screening and prioritization.

### Calibration and Public Health Utility (DCA)

The calibration analysis demonstrated high reliability in risk estimation, fulfilling the standards required for public health decision support frameworks. In children and adolescents, both models showed stable calibration; the SVM outperformed logistic regression in the 12-year cohort (Brier = 0.2326), while models adhered closely to the ideal diagonal in the 15–19-year group. The adult cohort (35–44 years) exhibited the highest reliability, with Brier scores reaching 0.2159 and decile-based analysis showing near-perfect alignment between predicted risks and observed frequencies. The public health utility of the models was assessed via DCA to determine the net benefit of AI-assisted screening compared to default strategies (treat-all and treat-none). Across all three cohorts (12 years, 15–19 years, and 35–44 years), the machine learning models demonstrated a consistent and positive net benefit across a wide range of threshold probabilities. In the adult group (35–44 years), the framework maintained a superior net benefit up to a risk threshold of nearly 0.8, significantly outperforming the universal screening approach. Similarly, for children and adolescents, utilizing the AI-driven triage resulted in higher gains at threshold probabilities between 0.1 and 0.6, indicating the framework is effective for population-level risk stratification and health equity planning.

### Predictor Importance and Explainability

The predictor importance analysis using SHAP values confirmed a hierarchical dominance of socio-structural factors over individual clinical markers across all studied age-groups (Fig. 1b). Wealth score and household density consistently emerged as the primary structural drivers of the disease burden throughout the lifespan. Explainability analysis revealed that the impact of these determinants is particularly pronounced when predicting caries severity (regression), where social vulnerability exhibits a direct, linear relationship with increased DMFT counts (Fig. 1b). In the 15–19 y cohort, for instance, lower wealth levels and higher household density were the factors with the greatest magnitude of positive impact on the predicted DMFT index (Fig. 1b). Across the three age cohorts, a shift in the relative contribution of predictors was observed: while socioeconomic status remained the dominant correlate of severity in every group, self-rated oral health status and service utilization patterns (time since last dental visit) emerged as important correlates of severity specifically in the 35–44-year cohort. As these findings derive from independent cross-sectional age-groups rather than repeated observations of the same individuals, this pattern should be interpreted as an age-related difference in correlates of severity rather than evidence of within-person change across the life course (Fig. 1b). The clinical and public health utility of these predictions was validated by DCA, which demonstrated a consistent positive net benefit when utilizing the AI models for population-level screening compared to “treat-all” or “treat-none” strategies (Fig. 1a, 2c).

## Discussion

The integration of AI and machine learning into cariology offers a transformative approach to addressing the global burden of dental caries [1, 8]. A key finding of this study is the consistent superiority of “white-box” models, such as logistic and negative binomial regressions, over complex ensemble methods [9, 30]. This suggests that within the large-scale epidemiological data of the SB Brasil 2023 [21], the relationship between socio-structural determinants and oral health outcomes is sufficiently captured by additive models [3, 4]. The explicit inclusion of race/color and socio-structural barriers ensures that machine learning serves to signal inequity rather than perpetuate it [14, 15]. This reinforces the argument that oral health disparities in Brazil are fundamentally driven by social determinants, which can be effectively mapped for population-level risk stratification without the need for high-dimensional “black-box” opacity [11, 34].

This transparency is a requirement for precision public health within the Brazilian National Health System (SUS) [7]. By providing clear coefficients for variables like household density, these models function as decision support frameworks for public health, allowing health managers to translate epidemiological data into actionable resource prioritization [9, 10]. The inclusion of a non-clinical triage model further enhances this utility, demonstrating that socio-behavioral and self-reported data can effectively identify high-risk individuals for prioritization even when clinical examinations are not immediately feasible [27]. Furthermore, the use of principal component analysis to generate the wealth score was pivotal; by reducing collinearity and condensing household assets into a single index, we ensured the AI captures a stable socioeconomic gradient across all age cohorts [26].

Our findings identify a marked shift in predictive signal during late adolescence (15–19 years), where socio-structural determinants explained a structural proportion (10.06%) of clinical variance [11, 19]. While an R2 of 10% might appear modest in clinical settings, in social epidemiology, it represents a meaningful structural signal that can effectively guide macro-level resource allocation [35, 36]. This age-cohort perspective suggests that late adolescence is a critical window where social risks translate most predictably into measurable clinical severity [19]. The convergence between caries presence and DMFT severity underscores how the social environment acts as a catalyst for both the initiation and progression of the disease [34]. Our framework thus maps the biological embodiment of social inequity, where structural vulnerabilities dictate disease patterns [37].

The discriminatory power achieved in adults is significant given the non-clinical nature of many predictors [16, 38]. In high-prevalence settings, the utility of these models lies in their ability to shift the probability of identifying high-risk groups rather than providing a definitive individual diagnosis [36]. By utilizing DCA, we demonstrated that our models provide a consistent net benefit compared to default “treat-all” or “treat-none” strategies, even when facing moderate negative predictive values [32]. This proves the framework’s ability to improve the logic of resource allocation, shifting the paradigm from purely clinical screening to a bio-social risk assessment that aligns with modern definitions of oral health [39]. Combined with low Brier scores and MAE values, these findings provide a reliable framework for AI-supported triage in public health dentistry [26, 31].

The mathematical robustness and interpretability of these algorithms pave the way for technological integration into managerial workflows [12, 13]. The proposed framework is suitable for the development of decision-support applications designed for health managers and public health planners [11, 12]. In the frontline of the National Health Systems, such tools could allow oral health teams to input simple socioeconomic data during initial community screenings to instantly identify high-risk clusters [11]. This implementation would facilitate a transition toward a data-driven triage system, enabling targeted interventions and more equitable resource allocation.

Despite its strengths, certain limitations must be acknowledged. First, the cross-sectional nature of the SB Brasil 2023 data prevents the establishment of causal links [21, 37]. Second, our study adopts an age-cohort perspective rather than a longitudinal life-course analysis; thus, we cannot assume that current adults experienced the same risk factors as today’s children in their youth [40]. Third, the survey focused exclusively on urban populations, potentially excluding unique rural risk factors [21]. Fourth, while we adhered to TRIPOD + AI [20] and PROBAST + AI [23] standards, our evaluation relied on internal validation. Although 5-fold cross-validation provides robust estimates, external validation remains a necessary step for future widespread implementation [38, 41]. Finally, while we identified key predictor weights, the absence of specific dietary or microbiological profiles in the national database might limit the models’ biological depth [16–18]. These limitations, however, do not undermine the utility of the models for public health risk stratification, provided they are interpreted within these defined boundaries.

The integration of machine learning into this framework goes beyond mere predictive automation; it addresses the nonlinear complexity and multidimensional interactions between social determinants that traditional aggregate measures often mask [35]. While community-level indicators (e.g., neighborhood-level income) are well established in the literature, they often assume a linear relationship that fails to capture intra-community heterogeneity [36]. Our machine learning approach identifies individuals whose specific bio-social profiles, integrating structural burden, household density, and self-rated oral health status, signal a heightened risk that would otherwise be invisible within community averages [27, 35]. Thus, the algorithm does not replace known variables; rather, it acts as a high-resolution sensor capable of detecting the synergy of vulnerabilities, providing a more granular basis for proportional universalism [36].

Furthermore, the utility of these models should not be framed as a response to resource scarcity in low-income settings but as a commitment to a bio-social and “upstream” approach to oral health [42]. By utilizing only non-clinical data in our triage models, we shift the focus to a “life-context” paradigm [39]. This allows for the identification of risk before it becomes irreversible, aligning with modern definitions of oral health that prioritize functional and psychosocial well-being [39, 42]. Our framework acts as a bridge between social epidemiology and precision health, using technology to map the structural “causes of the causes” and enabling health systems to intervene in the social gradient of the disease directly [3, 42].

This study demonstrates that AI provides a robust framework for population-level risk stratification, effectively translating complex epidemiological data into strategies for public health planning [1]. The identification of late adolescence as a strategic predictive window allows public health systems to implement upstream interceptive measures before significant damage accumulates [42]. Ultimately, the potential for deploying these algorithms provides a scalable solution for the managerial needs of the Brazilian National Health System (SUS), marking an essential step toward using technology to translate bio-social complexity into practical public health planning [12].

## Acknowledgments

The authors would like to thank the Ministry of Health, the Secretariat of Primary Health Care, the Department of Community Health Strategies and Policies, and the National Coordination for Oral Health Policy for their support and for providing the SB Brasil 2023 survey data.

## Statement of Ethics

In compliance with Resolution No. 466/2012 of the Brazilian National Health Council (CNS), SB Brasil 2023 was approved by the National Research Ethics Commission (CONEP) on July 3, 2021 (CAAE: 34497120.6.3001.0008; Opinion No. 4.823.054). Written informed consent was obtained from all participants and legal guardians of minors and vulnerable individuals. For participants under 18 years of age, written informed assent was obtained in addition to the consent signed by their legal guardians.

## Conflict of Interest Statement

The authors have no conflicts of interest to declare.

## Funding Sources

This study was not supported by any sponsor or funder.

## Author Contributions

J.M.A.G.: conceptualization, methodology, data curation, formal analysis, visualization, writing – original draft preparation, and project administration. O.L.A.J., M.L.B.F., and J.B.H.: formal analysis, validation, investigation, data curation, and writing – review and editing. F.N.H.: conceptualization, supervision, resources, and writing – review and editing. All authors have read and approved the final manuscript.

## Data Availability

The data that support the findings of this study are available from the Brazilian Ministry of Health. The SB Brasil 2023 dataset is available upon reasonable request to the National Coordination for Oral Health Policy (Secretariat of Primary Health Care). Microdata are publicly accessible through the official repository of the Ministry of Health at https://www.gov.br/saude/pt-br/composicao/saps/brasil-sorridente/sb-brasil/dados.
